# New record of a phoretic flea associated with earwigs (Dermaptera, Arixeniidae) and a redescription of the bat flea *Lagaropsylla signata* (Siphonaptera, Ischnopsyllidae)

**DOI:** 10.3897/zookeys.657.11095

**Published:** 2017-02-17

**Authors:** Michael W. Hastriter, Kelly B. Miller, Gavin J. Svenson, Gavin J. Martin, Michael F. Whiting

**Affiliations:** 1 Monte L. Bean Life Science Museum, Brigham Young University, 290 MLBM, P.O. Box 20200, Provo, Utah 84602-0200, USA; 2 Department of Biology and Museum of Southwestern Biology, University of New Mexico, Albuquerque, New Mexico 87131, USA; 3 The Cleveland Museum of Natural History, 1 Wade Oval Drive, Cleveland, Ohio 44106, USA; 4 Department of Biology, Brigham Young University, Provo, Utah 84606, USA

**Keywords:** *Arixenia esau*, Deer Cave, Gunung Mulu National Park, insect phoresy, *Lagaropsylla turba*, *Xeniaria jacobsoni*

## Abstract

*Lagaropsylla signata* (Wahlgren, 1903), previously known only from the Island of Java, Indonesia is redescribed and reported for the first time in Deer Cave, Gunung Mulu National Park, Sarawak, Malaysia (west coast of Borneo). Many were found clinging to the earwig *Arixenia esau* Jordan, 1909. A similar account of a phoretic flea (*Lagaropsylla turba* Smit, 1958) on the same species of cave-dwelling earwig has been reported in peninsular Malaysia in a well-documented association with the hairless naked bulldog bat, *Cheiromeles torquatus* Horsfield, 1824. The association of *Lagaropsylla signata* with *Arixenia esau* is parallel to the evolution and co-existence with bats in Deer Cave just as in the case of *Lagaropsylla turba*, *Arixenia esau*, and *Cheiromeles torquatus*. The evidence suggests that *Lagaropsylla turba* and *Lagaropsylla signata* are obligate phoretic parasites whose survival depends on *Arixenia esau* to access a bat host. *Arixenia esau* is reported for the first time in Deer Cave and the occurrence of *Lagaropsylla signata* on the island of Borneo represented a new record, previously being found only on the island of Java. Images of *Lagaropsylla signata* attached to *Arixenia esau* are provided. *Xeniaria jacobsoni* (Burr, 1912), often associated with *Arixenia esau* in other geographical areas, was not present in the material examined from Deer Cave. The natural history of the earwig genera *Arixenia* Jordan, 1909 and *Xeniaria* Maa, 1974 are discussed and summarized relative to their associations with phoretic fleas and their bat hosts.

## Introduction

Phoresy occurs in some insects and arachnids in which one species attaches to another species in a commensal relationship for the purpose of increasing their ability to disperse from one place to another. There is seldom a detrimental effect on the transporting host species. In some cases, attachment to another species is accidental, while others have evolved into vital components of their life history. Although there is an account of a bird flea attaching to a wasp (Rothschild & Clay, 1952), this was attributed to an accidental association of a wasp foraging on a flea-infested avian carcass. *Lagaropsylla turba* Smit, 1958 is the only documented species of flea that truly demonstrates phoretic behavior for which there was an association between a flea, another insect (*Arixenia esau* Jordan, 1909), and a bat host (the naken bulldog bat *Cheiromeles torquatus* Horsfield, 1824). In this scenario, an earwig provides a vehicle for *Lagaropsylla turba* to come into contact with its only known host, *Cheiromeles torquatus*. Nakakta & Maa (1974) provided a summary of the commensal forms of the dermapterid suborder Arixeniina and their associated molossid bat species. Important works cited by Nakata and Maa (1974) included: Jordan (1909), Jacobson (1912), Burr and Jordan (1913), Audey (1952), Cloudsley-Thompson (1957), Medway (1958), Giles (1961), and Popham (1962). Medway (1958) and Marshall (1977) described some crucial bionomical inter-relationships relative to *Lagaropsylla turba*, *Arixenia esau*, and *Cheiromeles torquatus*, while Marshall (1981) discussed similar parallel behaviors of co-existing associations between bird lice, hippoboscids, and their avian hosts. We reported herein a second flea species [*Lagaropsylla signata* (Wahlgren, 1903)] that has a similar phoretic association with *Arixenia esau*. We also redescribed *Lagaropsylla signata* and further documented a new locality for both *Lagaropsylla signata* and *Arixenia esau*.

## Materials and methods

Earwigs were collected from the floor of Deer Cave while conducting a general insect survey of Gunung Mulu National Park in October 2006 (04°01'18"N, 114°49'24"E) (KBM & GJS) and in January 2009 (04°02'32.8"N, 114°48'49.6"E) (GJS). Specimens were collected in the same location deep within the main gallery of the cave in the early evening. No earwigs were found in the entrance area and path through the first half of the cave, although mixed rock and guano piles were present. Approximately 1000 m and within sight of the “Garden of Eden doline” (a cave ceiling collapse that allows light to enter the main gallery), the path climbed an isolated, cone-shaped hill. Very few earwigs were present at the base of this hill, but numbers increased with elevation. The top of the hill, the observation path, and railings included earwigs. Thousands were present in October 2006 and only a few were present in January 2009. Individuals were observed actively walking, mating, and resting in small cracks. Males, females and nymphs were collected using forceps to overcome their strong grip on the surface of rocks, railings, and clothing. The greatest concentrations of individuals were at the highest points on the hilltop, which suggests a negative geotactic behavior. Individuals were not observed in the area around the base of the hill and nearby cave walls.

All specimens were collected into and stored in 95% ethanol and later examined in the laboratory. During microscopic examination, many fleas were noted attached to the earwigs. One species of earwig was present and the mouthparts and genitalia were dissected to facilitate and confirm our identification. Earwigs were photographed in ethanol with the aid of a Canon 6D DSLR camera and Visionary Digital Passport II imaging system. Image stacks were montaged with Zerene Stacker v.1.04. Fleas were mounted on microscope slides in accordance with procedures outlined by Hastriter and Whiting (2003:1043) and were illustrated with the aid of an Olympus BX61 Compound Microscope and an Olympus CC12 digital camera accompanied with an Olympus Microsuite B3SV program. All images were edited in Adobe Photoshop CC 2015. Earwigs and fleas were deposited in insect collections at the Monte L. Bean Life Science Museum, Brigham Young University, Provo, Utah (three *Arixenia esau*; MH-905: slides 7♂, 3♀ fleas, many fleas in alcohol), the Museum of Southwestern Biology, University of New Mexico, Albuquerque, New Mexico (99 *Arixenia esau*; MH-905: slides 2♂, 2♀ fleas,), and the Cleveland Museum of Natural History, Cleveland, Ohio (9 *Arixenia esau*; MH-905: slides 2♂, 2♀ fleas).

## Results

### Siphonaptera

#### Ischnopsyllidae

##### Ischnopsyllinae

###### Lagaropsylla signata

Taxon classificationAnimaliaSiphonapteraIschnopsyllidae

(Walgren, 1903)

Figs 1–2 , 3–6 , 7–9

####### Type species.

*Ceratopsylla signata* Walgren, 1903, Banjuwangi, Java, 22 V 1899, Carl Aurivillius, *Nyctinomus plicatus* [= *Chaerophon plicata* (Buchanan, 1800)] [number or sex of specimens in type series not recorded] (Swedish National Museum, Stockholm, not examined).

**Figures 1–2.**
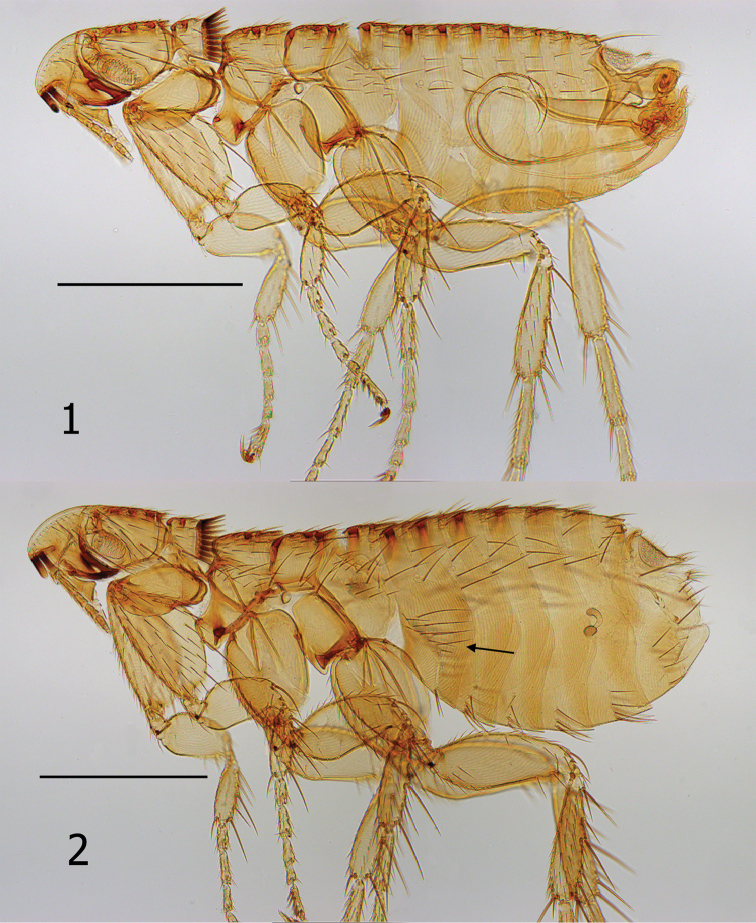
*Lagaropsylla signata* (MH-905). **1** Overview of male **2** Overview of female (arrow indicates group of setae on S-II). Scale bars: 0.2 mm.

**Figures 3–6.**
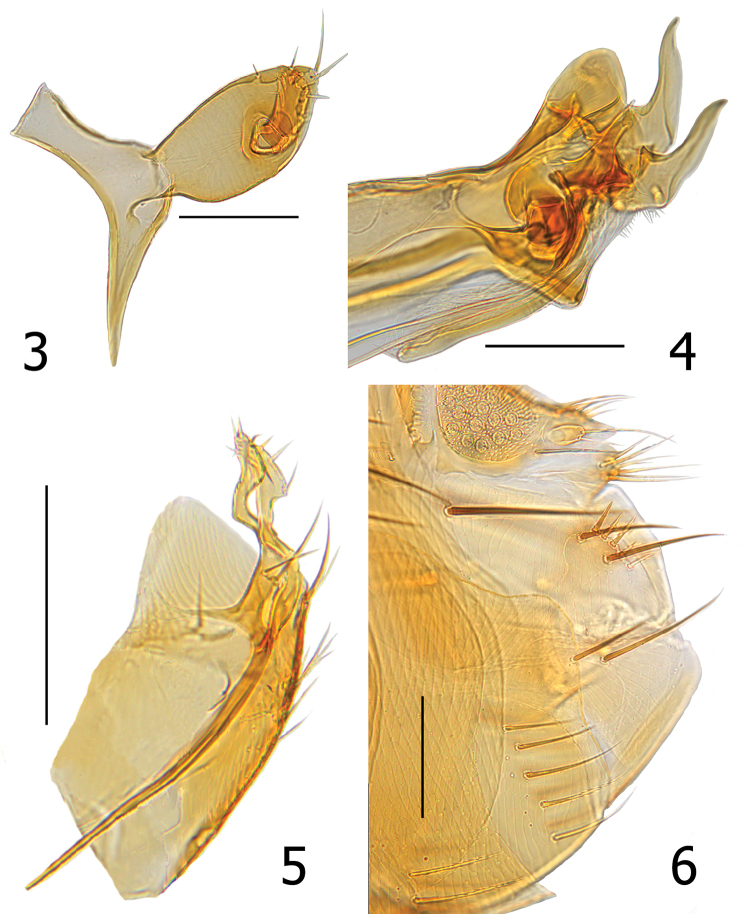
*Lagaropsylla signata* (MH-905). **3** Manubrium, basimere, and telomere, male **4** Terminal view of aedeagus, male **5** Sterna VIII and IX, male **6** Terminalia of female, illustrating T–VIII and S–VII. Scale bars: 0.1 mm (**3, 4, 6**), 0.2 mm (**5**).

**Figures 7–9.**
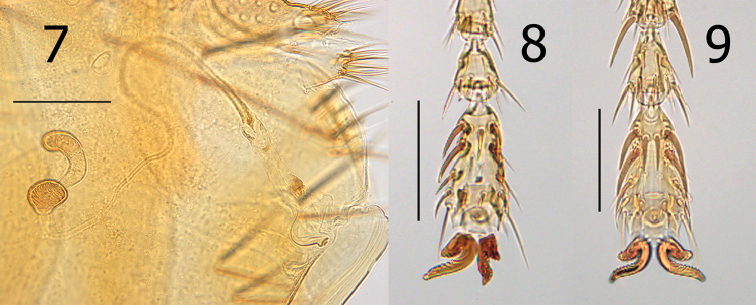
*Lagaropsylla signata*, female (MH-905). **7** Spermatheca and bursa copulatrix **8** Distitarsomere I **9** Distitarsomere III. Scale bars: 0.1 mm.

####### Diagnosis.

Both sexes may be distinguished from all other species of *Lagaropsylla* by a narrow band separating the margin of the frons from a white area anterior to the frontal row of fine setae. This band is hardly wider than that of the marginal layer of the frons (Figs 1–2). Closely allied to *Lagaropsylla mera mera* Jordan & Rothschild, 1921, males are easily separated by the position of the acetabulum on the basimere. The acetabulum is midway on the basimere of *Lagaropsylla signata* (Fig. 3) and far distal to the midline of basimere in *Lagaropsylla mera mera*. The broad concave lobe on the caudal margin of the female S-VII (Fig. 6) differs from all other species of *Lagaropsylla* Jordan and Rothschild, 1921.

####### Description.

Walgren (1903) provided only a brief description of *Lagaropsylla signata*. A detailed description is therefore provided to include illustrations of the species. Unless otherwise specified, counts of spines and setae are for one side of the flea.

Head. Frons evenly rounded with very thin band layered between margin and a broader white area caudad; caudal margin of white zone lined with a dozen small setae from oral angle to upper antennal fossa. Second genal tooth longer than first. Pre-oral tuber short and thick, only half the length of first genal tooth. Eye fused into upper heavily sclerotized margin of genal lobe, hardly discernible as distinct eye. Labial palpus of five short segments; sub-equal to length of maxillary palpus. Occipital area with dorsal incrassations (Figs 1–2).

Thorax. Length of pronotum equal to height of pronotum; 18 sharp ctenidial spines (both sides) equal to length of pronotum. One dorsal incrassation in pronotum; two dorsal incrassations in meso- and metanota. Prosternosome with antero-ventral area expanded ventrad. Pleural rod fused in center of sclerotic dome. Mesosternum and mesoepimeron fused as one; with two tuberculiform sclerotizations at juncture of sternum and epimeron. Pleural ridge feebly developed; pleural arch lacking. Lateral metanotal area dorso-ventrally flattened. Metepimeron with 12 setae in male and 21 or 22 in female (Figs 1–2).

Legs. Oblique suture of mesocoxa only indicated on ventral margin. Notch in metacoxa vestigial. All femora lacking lateral or mesal setation. Dorsal margin of all tibiae with six dorsal notches. Distitarsomeres each with five pairs of lateral plantar bristles; most proximal pair set onto plantar surface between second pair (Figs 8–9).

Unmodified abdominal segments. Abdominal terga I–VII each with a dorsal incrassation. Main rows of setae on T-I–VI interrupted; one seta below level of each spiracle. Spiracles round. One long antesensilial bristle. Sternum II of male without lateral setae. Female S-II with lateral patch of 14 setae; some short and others long and slender (Fig. 2). Both male and female with vertical parallel reticulations on S-II. Male without setae on S-III; one row of setae on S-IV–VII (2, 3, 3, 3). Female with one row of setae on S-III–VI (6, 4, 4, 4).

Modified abdominal segments, male. Saddle of T-IX and manubrium forming an obtuse angle. Basimere more convex on ventral margin than dorsal margin. Basimere with two or three small setae along dorsal margin; two moderately stout setae at apex. Acetabulum of telomere placed approximately midway between base and apex of basimere. Telomere half the length of basimere; slightly angled at ventral apical third terminating as acute angle at apex. Telomere with five or six minute setae along ventral margin; one minute seta at apex and two minute setae on dorsal margin (Fig. 3). Tergum VIII vestigial. Tendon of S-IX long; curved beyond and over apex of aedeagal apodeme (Fig. 5). Left and right halves of S-VIII fused along ventral margin; with dorsal lobe encompassing aedeagus. Two short and two longer setae at juncture of fused sclerites; lateral group of six or seven small setae (Fig. 5). Distal arm of S-IX (DA9) as in Fig. 5; apex with two setae, one slightly spiniform. Ventral margin of apical portion of DA9 with three minute marginal setae. Apex of proximal arm fused with base of aedeagal apodeme (fused area inseparable with dissection). Penis rods sub-equal in length to tendon of S-IX. Dorsal margin of aedeagal apodeme convex; apex acutely terminated. Dorsal spur present; fused with dorsal surface of T-IX. *Virga ventralis* thick but not darkly sclerotized. Crescent sclerite nearly vertical relative to longitudinal axis of aedeagal apodeme. Median dorsal lobe rounded; distal half more lightly pigmented than that of the surrounding area. Sclerotized inner tube shorter on ventral apical margin than dorsal margin; latter appearing in lateral view as fine, hair-like extension. Dorsal armature on dorsal margin of sclerotized inner tube thorn-like. Ventral surface with similar heavily sclerotized ventral armature. Crochet thin and tapering to apex; peg-like paxillus near ventral base of crochet (Fig. 4).

Modified abdominal segments, female. Tergum VIII with two small setae near spiracle VIII, three or four long lateral setae, and four or five mesal marginal setae (two stout, two or three fine). Sternum VIII tube-like without setae. Caudal margin of S-VII with broad, truncate lobe with slight concavity at middle; with vertical row of four setae. Anal stylet twice length of width; with two minute setae at base of two slender apical setae. Ventral apical seta not much longer than anal stylet; dorsal seta twice length of stylet (Fig. 6). Bulga of spermatheca longer than wide; cribriform area extended slightly beyond margin of bulga. Hilla narrowed at juncture of bulga and enlarged towards apex; apex usually twice width of base. Bursa copulatrix long; base with “C” shaped thin sclerite merging into long and broad duct of bursa copulatrix. Duct of bursa copulatrix with small sclerotizations but no associated sclerites outside of duct. Duct of spermatheca thickened and crinkled at exit of bursa copulatrix, narrowing towards spermatheca (Fig. 7).

####### Dimensions.

Male average length: 1.6 mm (n =10), range: 1.4–1.8 mm. Female average length: 1.7 mm (n = 7), range: 1.5–1.9 mm.

####### Material examined.

Malaysia, Sarawak, Deer Cave, Gunung Mulu National Park, 2♂, 3♀ attached to *Arixenia esau*, 15♂, 35♀ from bat guano, 6 X 2006, KBM. An additional 5♂ and 12♀ were removed from the bodies of five specimens of *Arixenia esau* collected in January 2009 by GJS.

### Dermaptera

#### Arixeniina

##### Arixeniidae

###### Arixenia esau

Taxon classificationAnimaliaSiphonapteraIschnopsyllidae

Jordan, 1909

Figs 10–11 , 12

####### Note.

**Figure 10–11.**
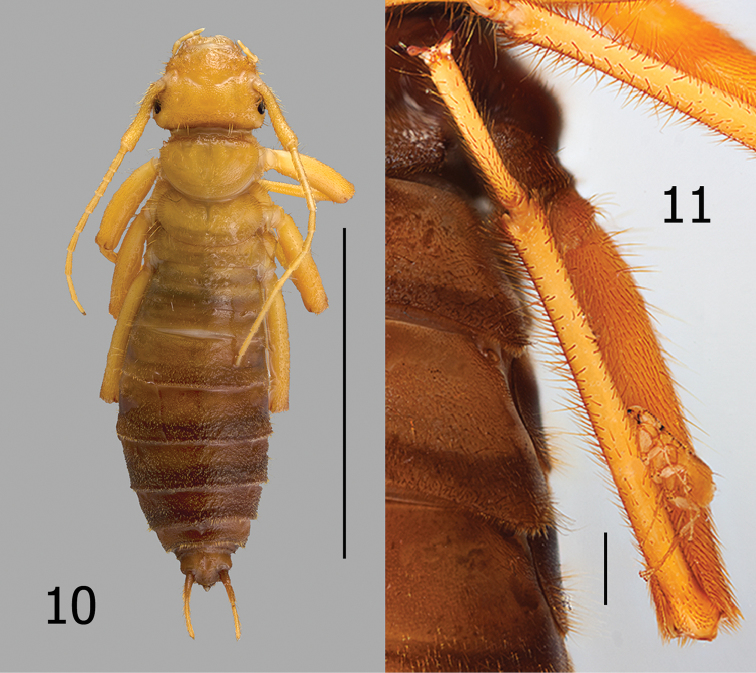
**10** *Arixenia esau*, female dorsal habitus, thought to be penultimate instar **11** Female *Lagaropsylla signata* attached to leg of female *Arixenia esau*. Scale bars: 10.0 mm (**10**); 1.0 mm (**11**).

**Figure 12.**
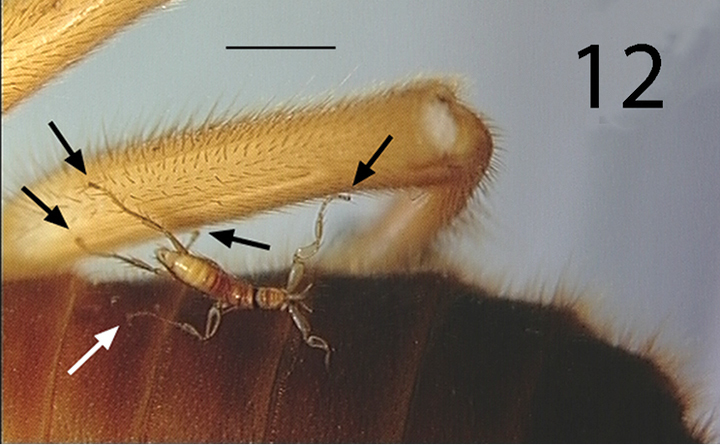
Male *Lagaropsylla signata* attached to hairs of hind femur and abdomen of female *Arixenia esau* (arrow indicate five points of attachment). Scale bar: 1.0 mm.

*Arixenia esau* is a robustly built, highly mobile earwig capable of transporting many fleas for significant distances (Fig. 10). Numerous specimens of *Lagaropsylla signata* were observed clinging to the hairs of *Arixenia esau*. Two such observations were photographed with the fleas in situ (Figs 11–12). The flea (Fig. 12) is grasping setae with five of its six tarsal claws (arrows). It was physically difficult to dislodge the fleas from *Arixenia esau* whose body and legs are somewhat densely covered with fine long setae (Figs 11–12).

####### Material examined.

*Arixenia esau*, Deer Cave, 162 m, Gunung Mulu National Park, 04°01'18"N, 114°49'24"E, Sarawak, Malaysia, 16 X 2006, KBM, (20 adult ♂, 45 adult ♀, 34 nymphs). *Arixenia esau*, Headquarters, 23 m, Gunung Mulu National Park, 04°02'32.8"N, 114°48'49.6"E, Sarawak Malaysia, 14–16, 21–25 I 2009, GJS, (2 adult ♂, 2 adult ♀, 5 nymphs).

## Discussion

*Lagaropsylla signata* was previously known only from the type series. Although the host was listed as *Chaerephon plicata*, no information of how the bat was collected (mist net), or its habitat (cave, open field, hollow tree, human dwelling, etc.) was provided. Banjuwangi, Java is only a general locality, since modern Banjuwangi is a sprawling metropolis at the eastern extreme of Java along the Straights of Bali. In our study, a total of 72 *Lagaropsylla signata* were collected from the bodies of *Arixenia esau* (7♂, 15♀) and from bat guano on the cave floor (15♂, 35♀). *Cheiromeles torquatus* was not found in Deer Cave in our study. Evidence of its historical presence was documented by Cranbrook (2010) in Niah Cave from archaeological records from the Pleistocene (40,000 years ago). Niah cave is approximately 115 km from Deer Cave. The finding of both *Lagaropsylla signata* and *Lagaropsylla turba* on *Arixenia esau* implies that they have both adopted the same phoretic vehicle to access a bat host. Such evidence suggests that *Cheiromeles torquatus* is present in Deer Cave and may serve as the principle host for *Lagaropsylla signata* but the definitive bat host for *Lagaropsylla signata* is unknown.

Because of the association of *Arixenia esau* (Fig. 10) and *Xeniaria jacobsoni* (Burr, 1912) (two closely related earwigs) and their potential bat hosts, a discussion of the known biology of each follows. Nakata and Maa (1974) and Marshall (1977) provided assessments of the dermapteran suborder Arixeniina and their associated molossid bats. The phylogenetic position of *Arixenia esau* as sister group to one lineage of free-living spongiphorid earwigs in the genus *Marava* Burr, 1911 has recently been proposed (Naegle, et al. 2016). Medway (1958) documented colonies of *Cheiromeles torquatus* in a “cave at Niah” (Subis Cave) in Sarawak, approximately 115 km from Deer Cave. From Subis Cave, Medway collected 23 fleas from a juvenile (non-flying) *Cheiromeles torquatus* that had fallen from the roost 250 feet above the chamber floor. He noted that fleas were also widespread on the floor and were often attached to living *Arixenia*. These fleas, plus two additional males of *Lagaropsylla turba* that were collected in a hollow tree in Ulu Gombak, Selangor, Malaysia, comprised the type series from which Smit described *Lagaropsylla turba*. A bat was not associated with the Ulu Gombak collection. *Arixenia esau* was documented on the bodies of *Cheiromeles torquatus* by Jordan (1909) and Medway (1958), while Cloudsley-Thompson (1957) reported “17 specimens of *Arixenia esau* from bats harvested from a durian (*Durio* sp.) tree cavity” in addition to 168 specimens roaming free within the same tree cavity. Nineteen specimens of *Xeniaria jacobsoni* were also reported by Cloudsley-Thompson (1957) from the same durian tree cavity, but none were collected from bats. Medway and Chong (1969) documented *Arixenia esau* on four mist-netted specimens of *Chaerophon plicata* in Pulai, Kelantan, and Borneo. Four specimens of *Xeniaria jacobsoni* were also reported by Burr (1912) from a cave near the shore of Babakan, Banjoumas Residency, Java. Further historic records of *Xeniaria jacobsoni* documented by Cloudsley-Thompson (1957) included two or three specimens from *Mops mops* (de Blainville, 1840) in Kuala Lumpur, Malaysia in July 1919, seven specimens from *Mops mops* in “Malaya” in 1920, an unspecified number from Mindanao, Philippines, and 35 specimens from South Java (Banjoemos) from cave near Babakan in 1933. The presence of two earwig species occurring in the same cave environments and both documented on different bat species remains an enigma. It appears that *Arixenia esau* is associated only with *Cheiromeles torquatus*, while *Xeniaria jacobsoni* has been found on *Mops mops*, *Chaerophon plicata*, and *Cheiromeles torquatus*. *Xeniaria jacobsoni* has been collected on only one occasion on the body of *Cheiromeles torquatus* but is found together with *Arixenia esau* in common environs. The discovery of multiple specimens of *Lagaropsylla signata* attached to *Arixenia esau* (behavior similar to *Lagaropsylla turba*) with no evidence of *Lagaropsylla signata* on any bat species will require further identification of bats in Deer Cave (and other caves) that may prove to harbor *Lagaropsylla signata*. The known distribution of bat hosts of Arixeniina species and the geographic distribution of *Lagaropsylla* spp. known from these areas are summarized in Table 1. *Arixenia esau* and *Xeniaria jacobsoni* have been associated in the hollows of trees but not in caves, while *Xeniaria bicornis* Maa, 1974 and *Arixenia camura* Maa, 1974 have been found together in the hollows of trees but neither have been found in caves (Nakata and Maa, 1974). The latter two species found only in Mindanao were reported by Nakata and Maa (1974) from *Chaerophon plicata* and *Cheiromeles torquatus*, respectively. Note: *Cheiromeles torquatus* does not occur in Mindanao, although *Cheiromeles parvidens*, a close relative, does. *Arixenia camura*, identified by Nakata and Maa (1974) would better be referred to *Cheiromeles parvidens* and not *Cheiromeles torquatus*.

**Table 1.** Records of *Arixeniina* taxa reported on bats and/or their environs by geographical localities (listed fleas apply only to localities, see footnotes).

**Localities and fleas**	**Dermaptera species**				
	***Arixenia camura***	***Arixenia esau***	***Xeniaria bicornus***	***Xeniaria jacobsoni***	***Xeniaria truncata***
Java				Cave	
Lagaropsylla signata*					
Malaysia, Peninsular		Hollow Tree		Hollow Tree	
Lagaropsylla mira**		*Cheiromeles torquatus*		*Cheiromeles torquatus*	
*Lagaropsylla turba*†		*Chaerophon plicata*			
Mindanao	“bats”		*Cheiromeles parvidens*		
	*Cheiromeles parvidens*		*Chaerophon plicata*		
	*Chaerophon plicata*?				
Palawan					*Cheiromeles torquatus*
Sabah, Malaysia		*Cheiromeles torquatus*			
Sarawak, Malaysia		Cave			
*Lagaropsylla signata*††		*Cheiromeles torquatus*			
*Lagaropsylla turba*‡					
Sumatra		*Cheiromeles torquatus*			

* *Lagaropsylla signata* reported from “*Tadarida plicatus*” = *Chaerophon plicata*. ** *Lagaropsylla mira* reported from *Chaerophon plicata* and *Mops mops*. Never phoretic. † *Lagaropsylla turba* collected from tree hole in Selangor State, Malaysia. †† *Lagaropsylla signata* collected from *Ariexenia esau* in Deer Cave, Gunung Mulu Nat’l Park. ‡ Lagaropsylla turba collected from *Arixenia esau* and from “young non-flying individual which had fallen to the cave floor” in Niah Cave, Niah National Park.

Medway (1958) observed negative geotropic behavior of *Arixenia esau*, many falling from associated roosting bats at heights 250 feet above, and that their principle foods were insects and glandular skin of *Cheiromeles torquatus*. Medway made no mention of earwigs on the cave walls. Burr and Jordan (1913) observed *Xeniaria jacobsoni* in countless numbers on the surface of guano and everywhere on the rocky walls at Gouwa Lawa (bat-cave), at Babakan, Java. This would suggest that *Xeniaria jacobsoni* (and likely *Arixenia esau* as well) could serve as the principle vehicle for fleas (developing in the guano on the cave floor, or in tree hollows) to ride to the roosting bats located high on the ceilings of huge caves. It is highly unlikely that adult fleas would be capable of making the journey from the cave floor to the bat hosts hundreds of feet overhead. In the massive caves that harbor *Cheiromeles torquatus*, these bats never make contact with the cave floor unless they fall to the floor as juveniles (which is fatal to the pups), or adults that bump into walls and are temporarily stunned. The inappropriate behavior of bumping into things (attributed to the presence of artificial light) was observed by Kirk-Spriggs (1989). Both earwig species feed on insects but *Xeniaria jacobsoni* feeds more voraciously on insects than does *Arixenia esau*, as their diet also includes cannibalism of their own kind, especially those that are vulnerable during molting (Burr and Jordan 1913). Popham (1962) studied the morphology of the mouthparts of both species and concluded that the mouthparts of *Arixenia esau* are more specialized for feeding (grazing) on the peculiar hairless, glandular exudates on the skin of *Cheiromeles torquatus*, whereas the mouthparts of *Xeniaria jacobsoni* are less specialized and more suited to feeding on insects (although it has been found on *Mops mops*, *Chaerophon plicata*, and *Cheiromeles torquatus*). The differences in the recorded occurrence of *Arixenia esau* and *Xeniaria jacobsoni* on different bat species may be a reflection of their differences in mouthpart morphology, dictating an ability to feed on the different skin (and hair) types.

Jordan (1909) made reference to *Arixenia esau* occurring in the sack formed by the membrane of the wings of *Cheiromeles torquatus*. Not to be confused with the gular pouch, Kitchener (1954) described the function of these pouches. With the wings inserted into these pouches (by manipulations of the hind legs and feet), they are neatly folded around the bat, facilitating a quadrupedal mode of mobility within their roost areas (caves, crevasses, and hollow trees). Schutt and Simmons (2001) also addressed the function of the sub-axillary pouches and their relation to quadrupedal mobility. *Arixenia esau* might have a lesser chance of falling from the highly active aerobatics of their bat hosts if they seek these protected areas during bat flight. According to Medway (1958), the gular pouch is not sufficiently large enough to accommodate *Arixenia*.

*Cheiromeles torquatus* is distributed in southern peninsular Thailand, Malaysia, the insular portions of Indonesia (Java, Sumatra, Borneo), and Palawan, Philippines (Schutt and Simmons, 2001). The distribution of *Arixenia esau* likely follows that of this bat host (although the distribution of *Arixenia esau* is less well defined than its host). Although *Cheiromeles torquatus* is listed in the IUCN Red List of Threatened Species, version 3.1, as “Least Concern”, studies and assessments are particularly urgent because it is rare in some areas of its range and destruction of many habitats (hollow trees in forested areas) are being destroyed along with logging and human encroachment. The distribution of *Xeniaria jacobsoni* appears to be much broader, but may be a reflection of greater collecting activities.

Both *Lagaropsylla turba* and *Lagaropsylla signata* appear to require earwigs to transport them to a viable bat host that would otherwise be inaccessible. Such obligate phoretic behaviors requires additional studies, especially to elucidate the association of *Lagaropsylla signata* and its yet unknown bat host species.

## Supplementary Material

XML Treatment for Lagaropsylla signata

XML Treatment for Arixenia esau
